# The extracellular endo-β-1,4-xylanase with multidomain from the extreme thermophile *Caldicellulosiruptor lactoaceticus* is specific for insoluble xylan degradation

**DOI:** 10.1186/s13068-019-1480-1

**Published:** 2019-06-08

**Authors:** Xiaojing Jia, Yejun Han

**Affiliations:** 1 Beijing Advanced Innovation Center for Food Nutrition and Human Health, Beijing Technology & Business University, Beijing, 100048 China; 2 National Key Laboratory of Biochemical Engineering, Institute of Process Engineering, Chinese Academy of Sciences, Beijing, 100190 China; 3 University of Chinese Academy of Sciences, Beijing, 100049 China

**Keywords:** Endo-β-1, 4-xylanase, Carbohydrate-binding module, Multi-modularity, Thermostability, Substrate specificity, *Caldicellulosiruptor lactoaceticus*

## Abstract

**Background:**

The extremely thermophilic bacterium *Caldicellulosiruptor lactoaceticus* can degrade and metabolize untreated lignocellulosic biomass containing xylan. The mechanism of the bacterium for degradation of insoluble xylan in untreated biomass has not been revealed.

**Results:**

In the present study, the only annotated extracellular endo-β-1,4-xylanase (Xyn10B) with multidomain structures in *C. lactoaceticus* genome was biochemically characterized. Xyn10B contains three N-terminal consecutive family 22 carbohydrate-binding modules (CBMs), one GH10 catalytic domain (CD), two family 9 CBMs and two S-layer homology (SLH) modules in the C-terminal. CBM22a shares 27.1% and 27.2% sequence homology with CBM22b and CBM22c, respectively. The sequence homology between two CBM9 s and two SLHs is 26.8% and 25.6%, respectively. To elucidate the effect of multiple domains on the enzymatic properties of Xyn10B, the truncated variants of which (Xyn10B-TM1: CBM22a-CBM22b-CBM22c-CD10; Xyn10B-TM2: CBM22c-CD10; Xyn10B-TM3: CBM22c-CD10-CBM9a; and Xyn10B-TM4: CD10-CBM9a) were separately reconstructed, recombinantly expressed and biochemically characterized. Enzymatic properties studies showed that the optimal temperature for all four Xyn10B truncations was 65 °C. Compared to Xyn10B-TM3 and Xyn10B-TM4, Xyn10B-TM1 and Xyn10B-TM2 had higher hydrolytic activity, thermostability and affinity on insoluble substrates. It is noteworthy that Xyn10B-TM1 and Xyn10B-TM2 have higher enzymatic activity on insoluble xylan than the soluble counterparts, whereas Xyn10B-TM3 and Xyn10B-TM4 showed opposite characteristics. The kinetic parameters analysis of Xyn10B-TM1 on xylan showed *V*_max_ was 5740, 1300, 1033, and 3925 U/μmol on insoluble oat spelt xylan (OSX), soluble beechwood xylan (BWX), soluble sugar cane xylan (SCX), and soluble corncob xylan (CCX), respectively. The results indicated that CBM22s especially CBM22c promoted the hydrolytic activity, thermostability and affinity on insoluble substrates of the Xyn10B truncations. The functions of CBM22, CBM9, CD and SLH are different, while contribute synergetically to the thermostability, protein structure integrity, substrate binding, and high hydrolytic activity on insoluble xylan of untreated lignocellulosic biomass. The domains of CBM22, CBM9, CD and SLH have different characteristics, which synergistically promote the thermostability, protein structure integrity, affinity on insoluble substrates and enzymatic activity properties of Xyn10B.

**Conclusions:**

The extracellular endo-β-1,4-xylanase with multidomain structures of CBM, CD and SLH promote the biodegradation of insoluble xylan in untreated lignocellulosic biomass by thermophilic *C. lactoaceticus*.

**Electronic supplementary material:**

The online version of this article (10.1186/s13068-019-1480-1) contains supplementary material, which is available to authorized users.

## Background

Lignocellulosic biomass, a source of renewable carbon for large-scale production of bio-fuels and platform chemicals, has been used in advanced bio-refineries. The polysaccharides of biomass have highly complicated constructions consisting of extensive and closely-knit networks of polymers which are recalcitrant to degradation [1]. To overcome this extraordinary challenge, microorganisms have created a large number of glycoside hydrolases to synergistically deconstruct polysaccharides [2].

Xylan, as one of the major polymeric hemicellulosic constituents in plant cell walls, is the second most prevailing biomass polysaccharide after cellulose in nature. It is a heteropolysaccharide consisting of a linear backbone chain of β-1,4-linked d-(+)-Xylose units which can be substituted by various residues, such as α-l-arabinosyl, *O*-acetyl, 4-*O*-methyl d-glucurono and feruloyl residues [3]. Complete deconstruction of xylan requires the synergism of xylanolytic enzymes. Among these enzymes, endo-β-1,4-xylanase (EC 3.2.1.8) plays a particularly key role since it randomly cleaves the β-1,4 glycosidic linkages in the long-chain xylan backbone, and produces short xylooligosaccharides which are further degraded into d-xylose by β-xylosidase (EC 3.2.1.37) [4]. According to the continually updated statistics from the Carbohydrate-Active Enzymes Database (CAZY, http://www.cazy.org/), endo-β-1,4-xylanase has been classified into glycoside hydrolase (GH) families 5, 8, 10, 11, 30, 43, 51 and 98, based on their physicochemical characters, structure and mechanism. Most endo-β-1,4-xylanase identified from bacteria, fungi, and plants belong to either GH10 or GH11. GH10 xylanases typically have higher molecular weight (> 30 kDa) with acidic isoelectric point (p*I*) as well as (β/α)_8_ barrel structure, whereas GH11 members generally process lower molecular weight less than 30 kDa with basic p*I* as well as β-jelly roll structure [4].

In addition to the diversity in biochemical functions, many hydrolases such as endo-β-1,4-xylanases evolved a complex modular architecture with one or more non-catalytic domains such as carbohydrate-binding modules (CBMs) and S-layer homology (SLH) [5]. According to the database of CAZY, CBMs have been classified into 84 families based on the amino acid similarity. CBMs are generally considered to potentiate the activity of their appended enzymes by bringing into close proximity with target substrate, in addition some CBMs have also been found to increase the thermostability of the enzyme [5]. CBM22 is a representative CBM that significantly promotes the thermostability of glycoside hydrolases, which was originally identified as non-catalytic thermostabilizing domains in xylanases from thermophilic bacteria [6, 7]. In addition, five conserved residues crucial for ligand binding were identified in the C-terminal CBM22-2 of *Clostridium thermocellum* Xyn10B by site-directed mutagenesis [8]. CBM9 is the only CBM found to date in xylanases, and has been proven to bind crystalline and amorphous cellulose, xylan, and xylooligosaccharides [9]. Besides CBM, many biomass-degrading bacteria are also facilitated by a diverse set of SLH domain-containing enzymes attaching on the bacterial surface [10]. Unlike CBM, which enhances enzyme activity and thermal stability, the extracellular enzymes bind to bacterial surfaces through their SLH domains, thereby promoting bacterial degradation of biomass [11]. *Caldicellulosiruptor lactoaceticus* 6A (DSM 9545) is an extremely thermophilic and anaerobic bacterium capable of degrading cellulose and xylan in untreated lignocellulosic biomass, the bacterium can grow in the range of 50–78 °C, the optimal temperature is 68 °C [12]. The untreated biomass was degraded into fermentable sugars by the synergism of intracellular and extracellular glycoside hydrolases of the microorganism [13]. To date, two intracellular endo-β-1,4-xylanases (Calla_1331 and Calla_1781) of *C. lactoaceticus* active on soluble xylan have been characterized [14]. However, the degradation process and enzymatic mechanism of *C. lactoaceticus* for insoluble xylan of lignocellulosic biomass have not been elucidated.

To understand the mechanism of untreated xylan degradation by *C. lactoaceticus*, the extracellular endo-β-1,4-xylanase was studied in present study. The predicted extracellular endo-β-1,4-xylanase was identified in the genome of *C. lactoaceticus*, which consisted three parallel family 22 CBM, one GH10 catalytic domain (CD), one tandem repeat of family 9 CBM domains and two triplicated SLH modules. Different truncated variants of the enzyme were engineered and characterized in order to study the role of each of the studied domains of the endo-β-1,4-xylanase of *C. lactoaceticus*. The characterization of the extracellular endo-β-1,4-xylanase will help to understand the mechanism of untreated biomass utilization by *C. lactoaceticus*.

## Results

### Cloning and sequence analysis of Xyn10B

**Fig. 1:**
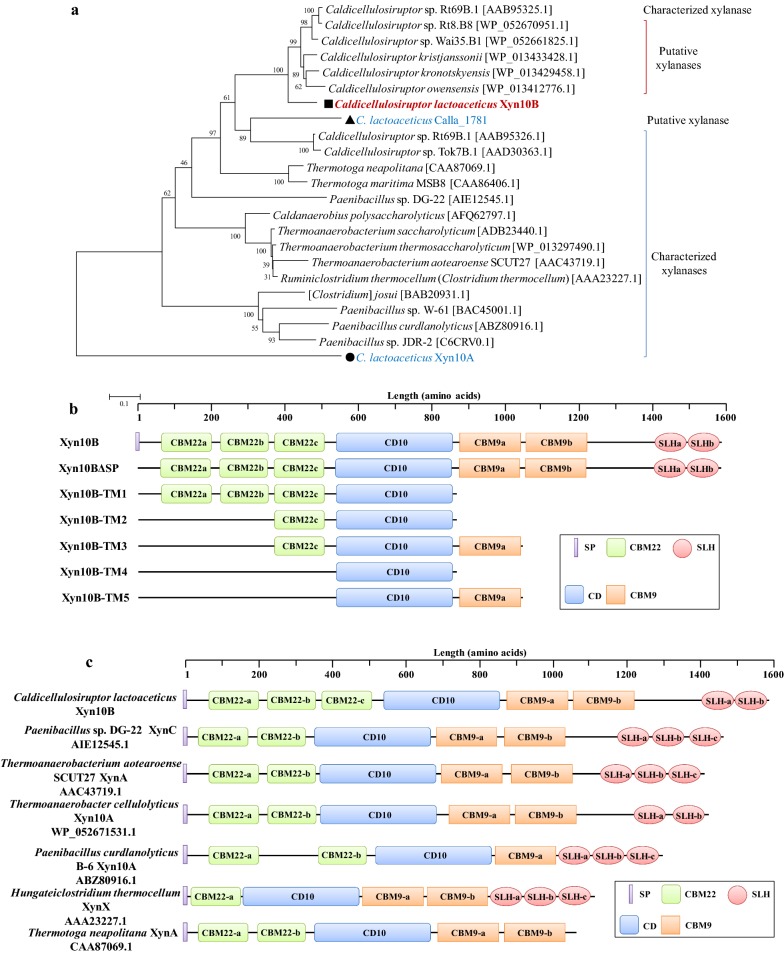
Phylogenetic tree analysis and modular structure of *C. lactoaceticus* Xyn10B. **a** Phylogenetic tree of *C. lactoaceticus* Xyn10B. The tree was constructed using MEGA 5.05 with 1000 bootstrap values, and GenBank accession numbers of each protein were listed after scientific names. **b** Schematic diagram of *C. lactoaceticus* Xyn10B domain structure. Xyn10BΔSP: Xyn10B without signal peptide; Xyn10B-TM1: CBM22a-CBM22b-CBM22c-CD10; Xyn10B-TM2: CBM22c-CD10; Xyn10B-TM3: CBM22c-CD10-CBM9a; Xyn10B-TM4: CD10; Xyn10B-TM4: CD10-CBM9a. **c** Modular structures of representative multidomain GH10 xylanases. The GenBank accession numbers of each protein were listed after scientific names. SP, signal peptide; CBM22, family 22 carbohydrate-binding module; CD10, family GH10 catalytic domain; CBM9, family 9 carbohydrate-binding module; SLH, S-layer-homology domain

Through *C. lactoaceticus* genome sequence analysis, Calla_0206 was annotated as a putative GH10 endo-β-1,4-xylanase (Xyn10B). SignalP analysis of the deduced amino acid sequence of Xyn10B confirmed the presence of an N-terminal signal peptide of 33 amino acid residues, suggesting that Xyn10B is a secreted protein. The gene *xyn10B* encodes a hypothetical protein of 1593 amino acids. Removal the signal peptide would result in a mature protein with a calculated molecular mass of 173.85 kDa and a theoretical p*I* of 5.07. BLAST analysis of the deduced amino acid sequence of Xyn10B showed that it had significant similarity with GH10 xylanases. Xyn10B had the highest 73% homology with endo-β-1,4-xylanase from *Caldicellulosiruptor* sp. Wai35.B1 [GenBank: WP_052661825.1], followed by 72% homology with endo-β-1,4-xylanase from *Caldicellulosiruptor* sp. Rt8.B8 [GenBank: WP_052670951.1] and *Thermoanaerobacter cellulolyticus* [GenBank: WP_052671531.1]. There are three annotated endo-β-1,4-xylanases in genome of *C. lactoaceticus*, i.e. Calla_1331, Calla_1781, and Calla_0206. The sequence homology of Xyn10B (Calla_0206) with Calla_1781 is 19.1%, and the sequence homology between Xyn10A (Calla_1331) and Xyn10B is 9.9%. The enzymes with high sequence similarity with Xyn10B were endo-β-1,4-xylanase from *Caldicellulosiruptor* sp. Wai35.B1 [GenBank: WP_052661825.1], *Caldicellulosiruptor* sp. Rt8.B8 [GenBank: WP_052670951.1] and *Thermoanaerobacter cellulolyticus* [GenBank: WP_052671531.1], while none of these enzymes had been characterized. In our previous work, both Calla_1781 and Calla_1331 (Xyn10A) were expressed in *E. coli*, whereas no activity was detected for Calla_1781, the intracellular xylanase Xyn10A was biochemically characterized [14]. Phylogenetic tree analysis clusters *C. lactoaceticus* Xyn10B with a significant number of relevant xylanases from named and deposited *Caldicellulosiruptor* species (Fig. 1a). In addition, it had a closer relationship with *C. lactoaceticus* Calla_1781 [GenBank: AEM74366.1], whereas showed distant relationship with *C. lactoaceticus* Xyn10A [GenBank: AEM73950.1].

On the basis of the conserved domain search and homology comparison in GenBank, mature Xyn10B is a multidomain enzyme composed of eight discrete domains (Fig. 1b): three N-terminal consecutive CBMs, one CD, two consecutive CBMs, and two consecutive SLH domains in the C-terminal. Similar endo-β-1,4-xylanase as Xyn10B also presents in other strains of *Caldicellulosiruptor* genus. The CD (L485-E817) is flanked by duplicated CBMs and shows high homology to catalytic domain of GH10. The highest homology (86%) of GH10 domain is found with xylanase from *Caldicellulosiruptor* sp. Wai35.B1 and Rt8.B8, and *T. cellulolyticus* [GenBank: WP_052661825.1, WP_052670951.1 and WP_052671531.1, respectively]. Multiple amino acid sequence alignment of the GH10 catalytic domain of *C. lactoaceticus* Xyn10B with other GH10 xylanases indicated that it is a typical (β/α)_8_ barrel structure, with Glu619, Glu734, and Trp790 in the active site (Additional file 1: Fig. S1).

Xyn10B has five CBMs which are found as discrete domains in GH enzymes and essential for insoluble substrate hydrolysis. The three CBM22 repeats (CBM22a, I8-S139; CBM22b, S159-A296; CBM22c, V319-T452) in the N-terminal non-catalytic region are different and homologous to non-catalytic thermostabilizing domain from other xylanases. The CBM22a shares 27.1% sequence homology with CBM22b, with 27.2% and 77.5% for that of CBM22a and CBM22c, CBM22b and CBM22c, respectively. CBM22a shows the highest homology of 73% with CBM22a of xylanase from *Caldicellulosiruptor kristjanssonii* I77R1B [GenBank: ADQ41707.1]. CBM22b displays the highest homology of 82% with *Caldicellulosiruptor kronotskyensis* xylanase [GenBank: WP_013429458.1] CBM22b domain. CBM22c has the highest homology of 72% with *C. kronotskyensis* xylanase [GenBank: WP_013429458.1] CBM22c domain. These CBM22 s in Xyn10B are shown to have about 57% to 82% sequence homology with CBM22 from genus *Caldicellulosiruptor*. The tandem CBM9 s (CBM9a, I837-M992; CBM9b, I1008-V1186), located at downstream of CD, share 26.8% sequence homology with each other. CBM9a has the highest homology (69%) with CBM9a domain of *Caldicellulosiruptor* sp. Wai35.B1 and *T. cellulolyticus* GH10 xylanase [GenBank: WP_052661825.1 and WP_052671531.1, respectively]. And CBM9b exhibits the highest homology (93%) with that of *Caldicellulosiruptor* sp. Rt8.B8 xylanase. The CBM9 s in Xyn10B are shown to have about 75% to 93% sequence homology with CBM9 from genus *Caldicellulosiruptor*.

Finally, two triplicated SLH domains (SLHa, Y1390-A1431; SLHb, F1506-E1547) at the C-terminal end function in anchoring the enzyme to cell surface in plant polysaccharide degradation. The SLHa shares 25.6% sequence homology with SLHb. And SLHa domain shows the highest 93%, as well as SLHb has the highest 100% homology with *C. kristjanssonii* I77R1B xylanase [GenBank: ADQ41707.1] SLH domain. The SLHs in Xyn10B are shown to have about 48% to 100% sequence homology with SLH from *Caldicellulosiruptor* sp.

### Expression and purification of recombinant Xyn10B truncations

As the size of Xyn10B (molecular mass of 173.85 kDa, 1593 amino acids) is large, it has not been successfully expressed in *E. coli*. To gain insight into the contribution of different domains to enzyme catalytic and binding activity, different truncated forms of Xyn10B were expressed and characterized. As the Xyn10B truncations will be recombinantly expressed in *E. coli* and purified, the N-terminal signal peptide and SLH domains were removed from the endo-β-1,4-xylanase. The truncations synchronously or intermittently contain CBM22, or CBM9, and CD10 were constructed. Truncations, namely Xyn10B-TM1 (CBM22a-CBM22b-CBM22c-CD10), Xyn10B-TM2 (CBM22c-CD10), Xyn10B-TM3 (CBM22c-CD10-CBM9a), Xyn10B-TM4 (CD10), Xyn10B-TM5 (CD10-CBM9a) were successfully cloned into pET28b vector. Recombinant proteins from crude extract of *E. coli* BL21 (DE3) were purified by Ni-affinity chromatography and determined by SDS-PAGE (Additional file 1: Fig. S2). The predicted molecular weights of Xyn10B-TM1, Xyn10B-TM2, Xyn10B-TM3, Xyn10B-TM4 and Xyn10B-TM5 were 91.0, 57.5, 73.6, 39.3, 55.4 kDa, respectively. Xyn10B-TM1, Xyn10B-TM2, Xyn10B-TM3 and Xyn10B-TM4 displayed as single band with molecular weight in agreement with the predicted sizes, Xyn10B-TM5 was not successfully expressed in soluble form for unknown reasons.

### Circular dichroism spectra (CDS) of Xyn10B derivatives

**Fig. 2:**
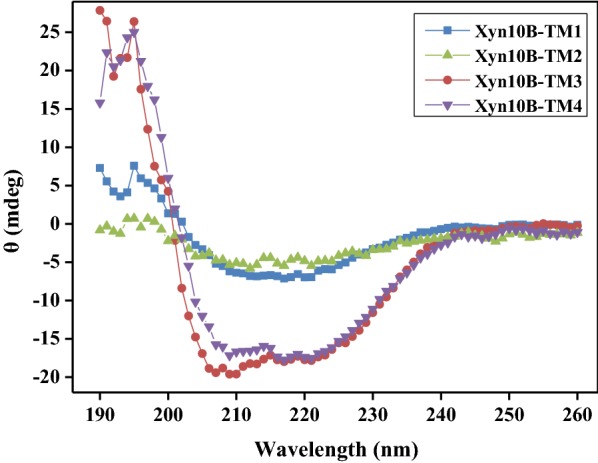
CD spectra of Xyn10B derivatives. The CD spectra for Xyn10B derivatives were performed on a JASCO J-810 spectrometer with protein concentration of 0.2 mg/mL in NaH_2_PO_4_ buffer (20 mM, pH 7.5). The CD spectra were measured at room temperature over a range of 190–260 nm using a quartz cuvette of 1 mm path length, with a bandwidth of 1 nm and a scanning speed of 100 nm/min. The measurements were conducted in triplicates, the data was corrected and analyzed by subtracting the buffer blank spectrum

The secondary structures of Xyn10B derivatives were analyzed with CDS. As shown in Fig. 2 and Additional file 2: Table S1, CD spectra showed a significant variance in their secondary structures. The content of α-helix for Xyn10B-TM3 and Xyn10B-TM4 was much higher than that of Xyn10B-TM1 and Xyn10B-TM2. The content of β-sheet for Xyn10B-TM1, Xyn10B-TM2, Xyn10B-TM3, and Xyn10B-TM4 was 32.5%, 25.7%, 0, and 25.6%, respectively. In addition, the content of random coil for Xyn10B-TM3 was 42.7%, which was higher than that of the other Xyn10B derivatives. The CD spectra analysis suggested that the secondary structures were influenced by different domain constitution.

### Biochemical characterization of the recombinant Xyn10B truncations

**Fig. 3:**
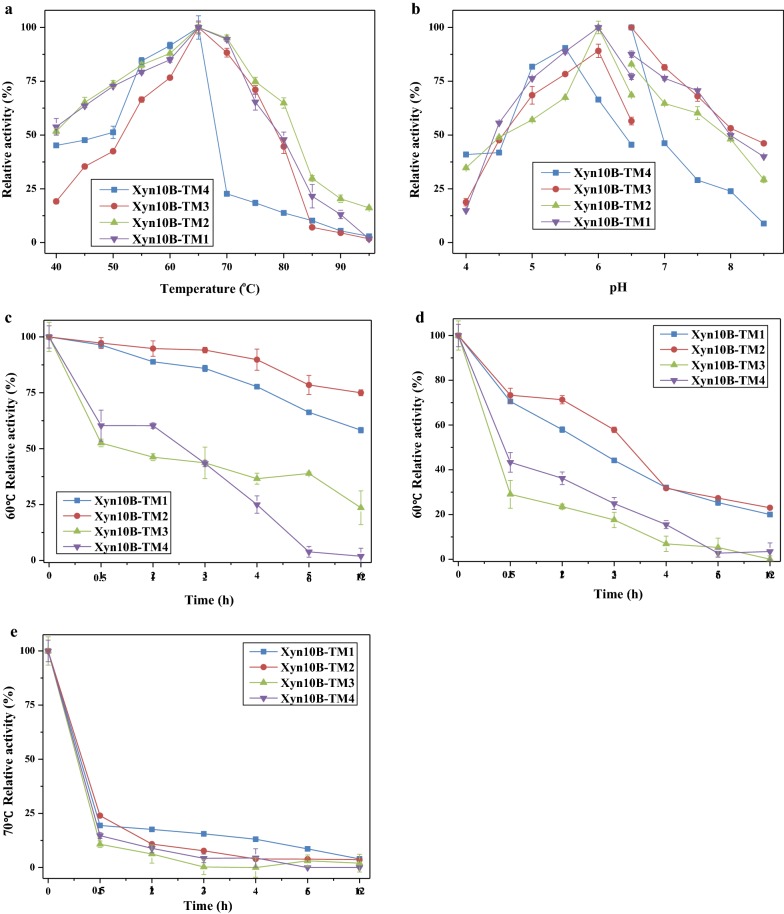
Temperature, pH and thermostability profile for Xyn10B-TM1, Xyn10B-TM2, Xyn10B-TM3 and Xyn10B-TM4. **a** Effect of temperature on the activities. **b** Effect of pH on the activities. **c** Effect of temperature on the thermostability

Using BWX as substrate, the optimal temperature of all the four truncations Xyn10B-TM1, Xyn10B-TM2, Xyn10B-TM3 and Xyn10B-TM4 was 65 °C (Fig. 3a). The optimal pH for both Xyn10B-TM1 and Xyn10B-TM2 were 6.0 in citrate buffer, and 6.5 in phosphate buffer for Xyn10B-TM3 and Xyn10B-TM4 (Fig. 3b). The results of thermostability assay showed that Xyn10B-TM2 had the highest thermostability, followed by Xyn10B-TM1, while Xyn10B-TM3 and Xyn10B-TM4 had much lower stability at elevated temperature (Fig. 3c). Xyn10B-TM1 and Xyn10B-TM2 lost thermostability quickly at 70 °C after 0.5 h. Xyn10B-TM3 lost nearly half of its activity at 60 °C after 0.5 h, and was almost inactive with incubation at 65 °C for 12 h. Xyn10B-TM4 had slightly better performance compared with Xyn10B-TM3. The stable temperature ranges for the truncated enzyme variants were influenced by the presence of different modules. Possessing CBM22c module resulted in a remarkably increase in thermostability of the catalytic domain (Comparison among Xyn10B-TM1, Xyn10B-TM2, and Xyn10B-TM4). However, the thermostability decreased when CBM22c and CBM9a presented together (Xyn10B-TM3) compared with Xyn10B-TM2. Notably, all of the four truncated variants retained only less than 24% of residual activity at 70 °C after 0.5 h, indicating that the truncated variants decreased the thermostability compared with the native enzyme.

Experiments were also carried out to study the specificity of Xyn10B truncated variants to various substrates. All of the four truncates showed high activity towards the polymer xylan, while no obvious catalytic activities were detected when Avicel, carboxymethyl cellulose, soluble starch, locust bean gum or chitosan were used as substrate. As shown in Table 1, Xyn10B-TM1 displayed the highest activities on BWX, SCX, OSX and CCX (944.3 ± 16.5, 837.0 ± 34.7, 5371.5 ± 281.1, and 3619.6 ± 84.7 U/μmol, respectively). The kinetic parameters *K*_m_ and *V*_max_ of Xyn10B-TM1 against xylan substrates were shown in Table 2. The activity of Xyn10B decreased with the removal of CBM22a and CBM22b on all of the four substrates. In contrast, the activity of Xyn10B-TM3 and Xyn10B-TM4 decreased sharply with all the xylan substrates compared with Xyn10B-TM1 and Xyn10B-TM2. Xyn10B-TM3, preserving the CBM22c and CBM9a domain, displayed the lowest activity on SCX. Meanwhile, Xyn10B-TM4 with only catalytic domain showed the lowest activity on BWX, OSX and CCX among Xyn10B truncated variants. Notably, CBM22 played critical role for the hydrolytic activity, while when CBM22c and CBM9a exist together, there will be negative effects.

**Table 1:** Specific activity of Xyn10B truncated variants with xylan substrates

Truncated variants	Specific activity^a^ (IU/μmol)			
	BWX	SCX	OSX	CCX
Xyn10B-TM1	944.3 ± 16.5	837.0 ± 34.7	5371.5 ± 281.1	3619.6 ± 84.7
Xyn10B-TM2	648.9 ± 10.3	600.1 ± 11.2	3720.4 ± 217.8	2046.6 ± 51.5
Xyn10B-TM3	457.5 ± 5.5	110.1 ± 20.4	1106.1 ± 65.0	1282.1 ± 79.3
Xyn10B-TM4	284.0 ± 15.5	111.6 ± 1.4	225.4 ± 31.8	229.1 ± 71.1

*BWX* Beechwood xylan, *SCX* sugarcane xylan, *OSX* oat spelt xylan, *CCX* corncob xylan

^a^The specific activity of each enzyme was determined under optimal conditions. ± indicates the standard deviation of three independent experiments

**Table 2:** Kinetic parameters of Xyn10B-TM1 with xylan substrates

Substrates	*V*_max_^a^ (IU/μmol)	*K*_m_^a^ (mg/mL)
BWX	1300.0 ± 82.8	4.2 ± 0.7
SCX	1033.0 ± 131.4	5.6 ± 1.6
OSX	5740.0 ± 303.8	1.2 ± 0.2
CCX	3925.0 ± 292.7	2.3 ± 0.5

*BWX* Beechwood xylan, *SCX* sugarcane xylan, *OSX* oat spelt xylan, *CCX* corncob xylan

^a^ The kinetic parameters of Xyn10B-TM1 against xylan substrates were determined in pH 6.0 citrate buffer at 65 °C. ± indicates the standard error of three independent experiments

### Binding properties of Xyn10B truncated variants on insoluble substrates

**Fig. 4:**
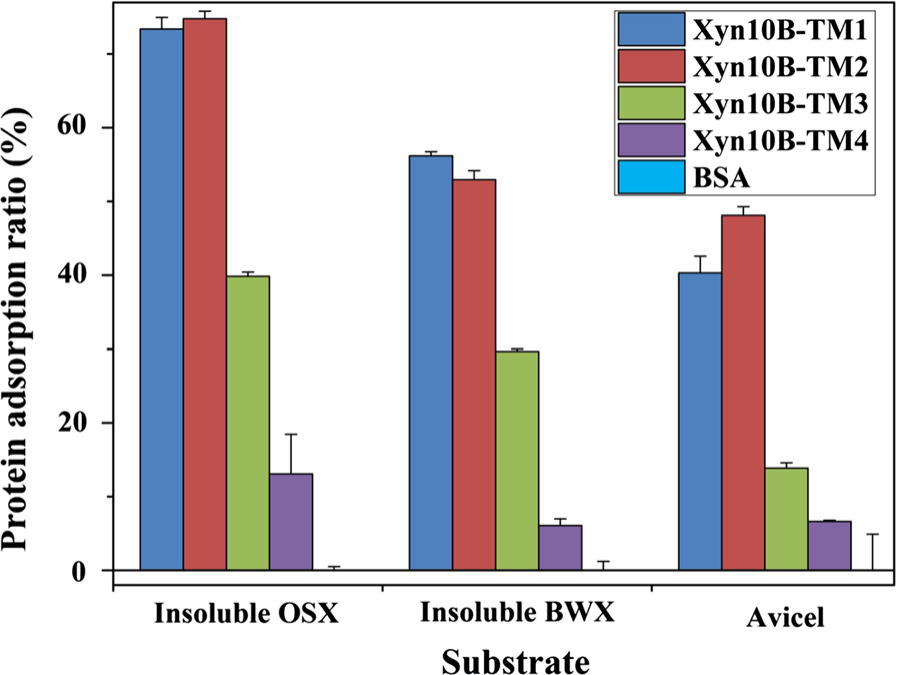
Binding ability of Xyn10B truncated variants on insoluble polysaccharides

The binding strength of the Xyn10B truncated variants to insoluble substrates was estimated by incubating the single enzyme with insoluble polysaccharides. As shown in Fig. 4, both Xyn10B-TM1 and Xyn10B-TM2 displayed similar affinity on the three insoluble substrates. Xyn10B-TM2 had the highest protein absorption towards insoluble OSX and Avicel, Xyn10B-TM1 displayed the highest affinity with insoluble BWX. The presence of CBM22c promoted the affinity towards insoluble substrates when compared with Xyn10B-TM4. In contrast, the affinity of Xyn10B-TM3 towards insoluble substrates decreased when CBM22c and CBM9a presented together. Additionally, Xyn10B-TM4 without any CBM also displayed slight binding activity with insoluble substrates. The binding properties of the four truncated variants on insoluble xylan were higher than those on Avicel.

### Hydrolytic properties of Xyn10B truncated variants

**Fig. 5:**
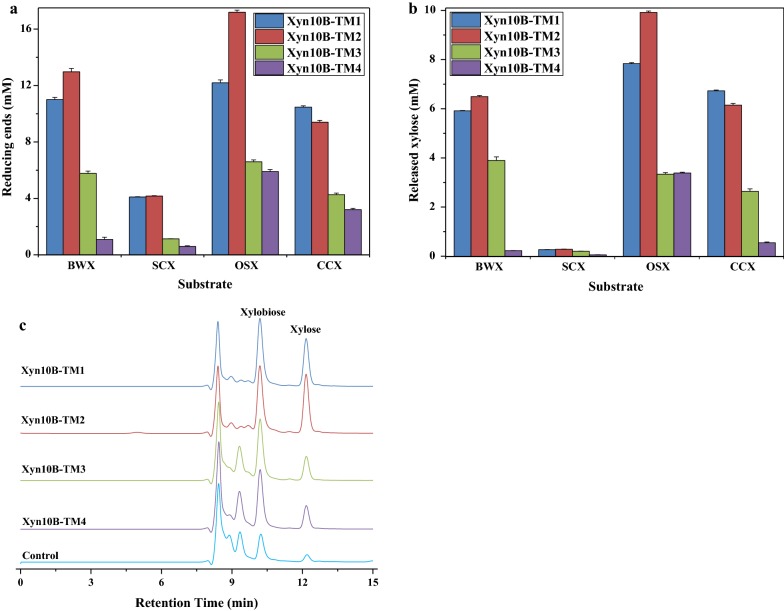
Hydrolytic patterns of Xyn10B truncated variants. **a** Reducing sugars produced by recombinant derivatives of Xyn10B. **b** Total xylose released from xylan. **c** HPLC analysis of the hydrolytic products of OSX

**Fig. 6:**
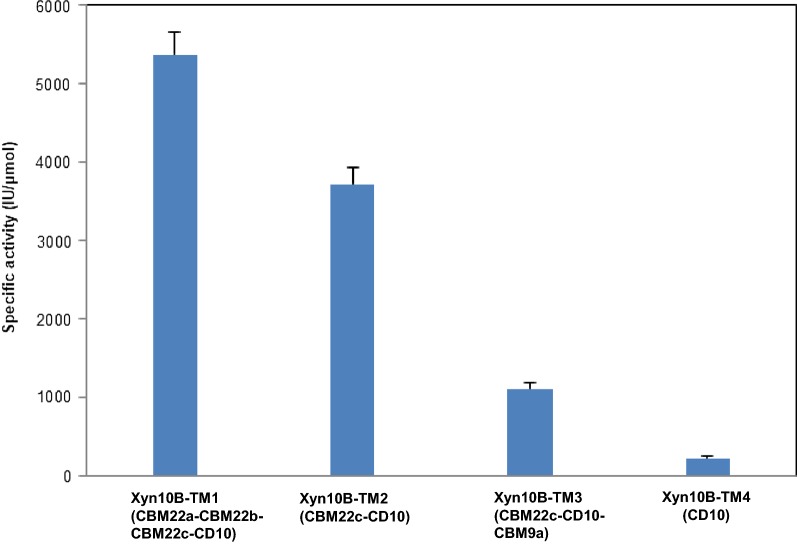
Specific activity of Xyn10B truncated variants on oat spelt xylan (OSX)

To determine the substrate hydrolysis patterns, Xyn10B truncated variants were separately incubated with various xylan containing substrates under optimum conditions. As shown in Fig. 5, Xyn10B-TM2 released the highest reducing sugars from BWX, SCX, and OSX, and Xyn10B-TM1showed the highest hydrolytic activity on CCX (Fig. 5a). Similar results were also observed in xylose production, Xyn10B-TM2 released the highest xylose from BWX, SCX, and OSX, and Xyn10B-TM1 produced the highest from CCX (Fig. 5b). The HPLC assay suggested that xylose and xylooligosaccharides were released by all of the four Xyn10B truncated variants (Fig. 5c). Moreover, the hydrolytic activity of the four Xyn10B truncated variants towards insoluble OSX was much higher than those soluble substrates. The activity of Xyn10B-TM2 versus insoluble OSX was higher than all of the other truncated variants. In addition, the results suggested that CBM22 promoted the hydrolytic ability of Xyn10B truncated variants, while CBM9a decreased which when presented together with CBM22c (Fig. 6).

## Discussion

Screening of the whole genome indicated that *C. lactoaceticus* has three xylanase genes. This multiplicity of xylanases occurs due to the heterogeneous nature of xylan polymer and is common in microorganisms [15]. To deal with the heterogeneous complexity of xylan, microbes produced certain specialized xylanases, which possessed different enzymatic property and substrate specificity. In the previous study, we characterized the two intracellular xylanases (Calla_1331, namely Xyn10A and Calla_1781) [14]. To investigate the xylanolytic system of *C. lactoaceticus*, the only extracellular xylanase Xyn10B was cloned, expressed, and characterized in the present study. The Xyn10B shared higher homology with Calla_1781, whereas lower homology with Calla_1331 (Xyn10A). Comparing the molecular architecture, both Calla_1781 and Xyn10B contained a CBM22 tandem domain, while the former was predicted as an intracellular enzyme. Homology alignment revealed that Xyn10B from *C. lactoaceticus* was a multidomain xylanase classified in GH family 10. Xyn10B is a secreted xylanase with high molecular weight (173.85 kDa) and acidic p*I* (5.07) which agrees with the characters of GH10 family. BLAST analysis and phylogenetic tree analysis also group Xyn10B as GH10 xylanases, which has a (β/α)_8_ barrel structure with two glutamate residues (Glu619 and Glu734) as catalytic residues. The architecture of CBM22/GH10/CBM9/SLH was found in a subset of GH10, whereas the domain varies significantly across species (Fig. 1c). The multidomain GH10 xylanases were characterized with two or three CBM22 located in the N-terminal region, followed by a GH10 catalytic domain, then accompanied with one or two CBM9 domains, and the C-terminal region is consisted of up to three SLH domains [16–20]. The multidomains contribute to the specific function of xylanases, which has been testified in *Paenibacillus barcinonensis* Xyn10C with CBM22/GH10/CBM9 architecture [21].

In general, the hydrolysis of insoluble polysaccharide, e.g. insoluble xylan, cellulose, etc., requires GH to approach and anchor to the sugar chains. CBMs potentiate the enzymatic deconstruction of pectic and hemicellulosic polysaccharides in both primary and secondary cell wall structures by targeting and proximity effects [22]. Previous crystal structure study showed extensive surface contacts between the catalytic module and CBM of *Clostridium thermocellum* Xyn10B [23]. However, investigations about the effects of CBMs on the thermostability and catalytic efficiency of multidomain xylanases have not come to unified conclusions [24]. Truncated derivatives of multidomain GH10 xylanases displayed distinct behavior with the absence of N- or C-terminal non-catalytic CBM modules. For example, truncated individuals of *Thermotoga maritima* MSB8 GH10 xylanase rXTMA had obviously different optimal temperature range, thermostability, and specific activities towards xylohexaose and insoluble polymeric heteroxylan [25]. The C-terminal CBM9-2 of xylanase 10A from *T. maritima* bound specifically to the reducing ends of both cellulose and soluble polysaccharides [26]. However, Fusion the N-terminal parallel CBM22 s of *Thermotoga neapolitana* Xylanase A to *Bacillus halodurans* S7 GH10 xylanase decreased the thermostability but enhanced the hydrolytic efficiency against insoluble xylan [27].

In glycoside hydrolase with multidomains, Family 22 CBM was often found together with GH10 catalytic module. Originally, CBM22 was identified and specified as a thermostabilizing module since the removal of which caused obvious reduction in the thermostability and/or optimum temperature of enzymes in both thermophilic and mesophilic bacteria [6, 7]. CBM22 were found to bind a large range of soluble and/or insoluble substrates including xylan [28]. According to CAZY, CBM family 9 has affinity with many substrates including amorphous and crystalline cellulose, xylan, and small soluble sugars such as cellobiose, cellooligosaccharides, and xylooligosaccharides. However, the CBM9 of *T. maritima* xylanase 10A did not have any obvious binding activity due to the lack of certain conserve residues involved in interacting with ligand [9].

In the present study, the characterization of Xyn10B suggested that the enzyme is an specific endo-β-1,4-xylanase for degrading insoluble xylan (Fig. 1c). The truncated derivatives with different module assembly mode displayed notable variation in thermostability, hydrolytic activity and substrate specificity. CBM22s of Xyn10B promoted both thermostability and affinity towards both insoluble xylan and Avicel. Removal of the CBM22s results in decrease of insoluble substrates affinity, thermostability and partial loss of catalytic activity. The addition of a CBM22 to *Clostridium stercorarium* Xyn10B drastically shifted the optimum temperature and enhanced the catalytic activity towards xylan. In the present work, compared with Xyn10B-TM2 (CBM22c-CD10), truncating a second ancillary CBM9 in Xyn10B-TM3 (CBM22c-CD10-CBM9a) incontrovertibly caused considerable reduction in thermostability and enzymatic activity. The results suggested the different function of CBM22 and CBM9. Further analysis by CD spectra suggested that CBM22c the correct folding of the secondary structure of Xyn10B trunctions. Tandem CBMs were also found to have different performance for substrate specificity, the N-terminal parallel CBM22s from *T. neapolitana* xylanase A showed xylan binding ability while not on cellulose [27]. CBM22 of *Paenibacillus* sp. strain W-61 GH10 xylanase Xyn5 bound to xylan, whereas CBM9 bound to Avicel [16].

The enzymatic properties of Xyn10B truncated variants were compared with other thermophilic xylanases with similar domain characteristic enzyme (Table 3). The specific activities of *C. lactoaceticus* Xyn10B-TM1, Xyn10B-TM2, Xyn10B-TM3, and Xyn10B-TM4 on OSX were 5371.5, 3720.4, 1106.1, and 225.4 IU/μmol, respectively. The specific activities of *Thermoanaerobacterium aotearoense* SCUT27 XynAΔSLH (A1-A2-B-C1-C2), *T. saccharolyticum* NTOU1 XynA (A1-A2-B-C1-C2-D1-D2-D3), alkaline wastewater sludge Xyn-b39 (A1-A2-B-C1-D1-D2-D3), *T. saccharolyticum* B6A-RI XynA (A1-A2-B-C1-C2), *Bacillus* sp. BP-23 xylanase C(A1-A2-B-C1-C2), *T. neapolitana* XynA (A1-A2-B-C1-C2) on OSX were 24159.4, 22022.0, 2464.0, 29562, 12.06, and 12910.8 IU/μmol [29–34].

**Table 3:** Comparison of properties between *C. lactoaceticus* Xyn10B truncated variants and other thermophilic xylanases

Xylanases from microorganism	Domain^a^	Mw (kDa)^b^	Topt (°C)	pHopt	Specific activity (IU/μmol)		References
					BWX	OSX	
*C. lactoaceticus* Xyn10B-TM1	A1-A2-A3-B	91.0	65	6.0	944.3 ± 16.5	5371.5 ± 281.1	This study
Xyn10B-TM2	A3-B	57.5	65	6.0	648.9 ± 10.3	3720.4 ± 217.8	This study
Xyn10B-TM3	A3-B-C1	73.6	65	6.5	457.5 ± 5.5	1106.1 ± 65.0	This study
Xyn10B-TM4	B	39.3	65	6.5	284.0 ± 15.5	225.4 ± 31.8	This study
*Thermoanaerobacterium aotearoense* SCUT27 XynAΔSLH	A1-A2-B-C1-C2	113	80	6.5	42,917.4 ± 361.6	24159.4 ± 180.8	[20]
*T. saccharolyticum* NTOU1 XynA	A1-A2-B-C1-C2-D1-D2-D3	154	72	5.5	66836.0 ± 2464	22022.0 ± 1078.0	[34]
Alkaline wastewater sludge Xyn-b39	A1-A2-B-C1-D1-D2-D3	160	60	7.0	6588.8 ± 384	2464.0 ± 272	[35]
*Thermotoga thermarum* Xyn10A	A1-A2-A3-B-C1-C2	131	95	7.0	19101.11	NA	[36]
*T. saccharolyticum* B6A-RI XynA	A1-A2-B-C1-C2	130	70	5.5	NA	29562	[37]
*Bacillus* sp. BP-23 xylanase C	A1-A2-B-C1-C2	120.6	45	5.0	NA	12.06	[38]
*T. neapolitana* XynA	A1-A2-B-C1-C2	116	102	5.5	NA	12910.8	[39]

*Xyn* Xylanase, *Mw* molecular weight, *Topt* optimum temperature, *pHopt* optimum pH, *BWX* beech wood xylan, *OSX* oat spelt xylan, *NA* not available

^a^A1, A2, A3, B, C1, C2, D1, D2 represents the N and/or C-terminal domains, A1, A2, A3: N-terminal family 22 CBM; B: catalytic domain; C1, C2: C-terminal family 9 CBM; D1, D2, D3: C-terminal SLH

^b^Value by SDS-PAGE

Generally, CBMs were found in GHs that primarily degraded complex carbohydrates to sugars [5]. Many bacteria and archaea produce surface layer proteins which are non-covalently associated with the bacterial cell surface via specialized SLH domain [10]. In the cellulosomal bacteria, SLH proteins contributes to multi-enzyme cellulosome complex adhesion and possibly hydrolysis [35]. Lots of non-cellulosomal biomass-degrading bacteria produce multidomain SLH domain-containing GHs for lignocellulose deconstruction [10]. However, SLH domains neither contribute to enzymatic activity nor are necessary for substrate binding [36]. It has been widely observed that *Caldicellulosiruptor* species secrete free and S-layer-bound cellulases and hemicellulases with the absence of cellulosomes [10, 13, 37]. The two triplicated SLH domains at the C-terminus of Xyn10B may also have the same function and contribute to biomass attachment. It had been believed that the constitutively excreted enzymes liberated small xylooligosaccharides which could be further transported into the cell and degraded by intracellular β-xylosidase or endo-β-1,4-xylanase [4]. The complexity of the domain structure guarantees the efficiency of cell-substrate adhesion, binding, and extracellular hydrolysis of Xyn10B with high polymer xylan.

## Conclusions

The extremely thermophilic bacterium *Caldicellulosiruptor lactoaceticus* has evolved special glycoside hydrolases to degrade untreated lignocellulosic biomass including the insoluble xylan. In our previous work, the thermophilic intracellular endo-β-1,4-xylanase for soluble xylan and xylooligosaccharides degradation has been studied. To understand the mechanism of degradation of insoluble xylan, the only annotated extracellular GH10 endo-β-1,4-xylanase Xyn10B with multidomains identified in the *C. lactoaceticus* genome was biochemically characterized. Studies on Xyn10B trunctions have found that CBM22s of Xyn10B promoted the hydrolytic activity, thermostability and substrate binding on insoluble xylan. The functions of the domains of CBM22, CBM9, CD, SLH are different, while contribute synergetically to the thermostability, protein structure integrity, substrate binding, and hydrolytic activity. The characteristics of extracellular Xyn10B will help to elucidate the mechanism of insoluble xylan and untreated lignocellulosic biomass degradation in thermophilic *C. lactoaceticus*.

## Materials and methods

### Strains, plasmids and chemicals

The strain *C. lactoaceticus* DSM 9545 was obtained from DSMZ (Braunschweig, Germany), and used for genomic DNA extraction. *Escherichia coli* Top10 (TianGen, China) and plasmid pET-28b (Novagen, USA) were used for gene cloning and sequencing. The *E. coli* strain BL21 (DE3) and Rosetta (DE3) (Novagen, USA) were used for further protein expression. Beechwood xylan (BWX, xylose 81.3%, glucuronic acid 13.0%, other sugars 5.7%, solubility in water 2.18 g/L) was purchased from Sigma-Aldrich (St. Louis, USA). Oat spelt xylan (OSX, xylose 70%, arabinose 10%, glucose 15%, solubility in water < 0.2 g/L) was obtained from Hualan Chemical Technology Co., Ltd. (Shanghai, China). Sugarcane xylan (SCX, xylan 72.5%, glucose 2.2%, arabinose 11.5%, uronic acid 13.3%, the solubility of SCX in water is 0.77 g/L.) and corncob xylan (CCX, xylan 85.5%, glucose 2.8%, arabinose 7.6%, galactose 4.2%, uronic acid 7.3%, solubility in water > 3.0 g/L) were prepared through the combination of alkaline and enzymatic hydrolysis by Professor Hourui Zhang from Guangxi Institute of Botany, Chinese Academy of Sciences [38, 39]. d-Xylose, Avicel, carboxymethyl cellulose, soluble starch, locust bean gum, chitosan, and other chemicals for buffer preparations were purchased from Kepujia Reagent Co. (Beijing, China). All other chemicals were of analytical grade unless otherwise stated. Genomic DNA extraction and truncated variants amplification.

*Caldicellulosiruptor lactoaceticus* 6A cells were cultured anaerobically in DSMZ 640 liquid medium at 70 °C for 72 h, and then the genomic DNA was extracted using a TIANamp Bacteria DNA Kit (TianGen, Beijing, China). Gene encoding a multidomain endo-β-1,4-xylanase Xyn10B [GenBank: AEM72886.1] was predicted and truncated derivatives (Fig. 1b) were amplified based on the whole genome. Primers used were as follows: Xyn10B-F1: 5′-GCCGCGCGGCAGCATGTTTTGAAAGCGGTAATC-3′, Xyn10B-F2: 5′-GCCGCGCGGCAGCATGTGTAAAATCAGCGACATTTG-3′, Xyn10B-F3: 5′-GCCGCGCGGCAGCATGACTCTTTGAGCAGTATAC-3′, Xyn10B-R1: 5′-GCGGCCGCAAGCGTTTATGACGGTTCAACAATTGCC-3′, Xyn10B-R2: 5′-GCGGCCGCAAGCGTTTAATTGTCAAAGATTGCTACATC. Among them, pairing primers F1 and R1 were used for cloning gene *xyn10B*-*TM1*, with F2 and R1 for *xyn10B*-*TM2*, F2 and R2 for *xyn10B*-*TM3*, F3 and R1 for *xyn10B*-*TM4*, F3 and R2 for *xyn10B*-*TM5*, respectively. PCR reactions were performed with Pfu DNA polymerase (TianGen) and the procedures were listed below: a hot start at 95 °C for 5 min, 30 cycles consisting of denaturation at 95 °C for 30 s, annealing at 55 °C for 1 min, and extension at 72 °C for 1 min. Finally, all the amplifications were maintained at 72 °C for 5 min and then cooled to 10 °C. Target PCR products were then purified using TIAN gel Midi Purification Kit (TianGen) and stored at − 20 °C until use.

### Construction and sequencing of the expression vector

Purified PCR products of truncated variants were separately treated with T4 DNA polymerase (Takara, Dalian, China) at 37 °C for 30 min, accomplished with inactivation at 75 °C for 20 min. After that, the treated insert genes were inserted into pET-28b EK/LIC vector at 22 °C for 20 min and then transformed respectively into *E. coli* Top10 competent cells and grown overnight on Lysogeny Broth (LB) agar plates with kanamycin (50 μg/mL) at 37 °C. Positive recombinant plasmids were finally confirmed by colony PCR and DNA sequencing.

### Expression and purification of the recombinant Xyn10B truncated variants

Recombinant plasmids were extracted using TIANprep Mini Plasmid Kit (TianGen) and transformed individually into *E. coli* BL21 (DE3) or Rosetta (DE3) competent cells and cultured overnight on solid LB medium with kanamycin (50 μg/mL) or combination of kanamycin and chloramphenicol (50 μg/mL each) at 37 °C. Single colonies were picked from the plates, and inoculated overnight in liquid LB medium with kanamycin (50 μg/mL) at 220 rpm and 37 °C, respectively. Cultures were grown individually in LB medium containing kanamycin (50 μg/mL) with the ratio 1:100 (v/v) at 37 °C with shaking at 220 rpm until the optical density at 600 nm reached approximately 0.6. Expression was induced with 0.5 mM isopropyl-β-d-thiogalactopyranoside (IPTG) and cells were further cultured for an additional 6 h at 37 °C. Cells were harvested by centrifugation (4000×*g*, 15 min, and 4 °C), and resuspended in binding buffer (50 mM Tris–HCl, pH7.5, and 300 mM NaCl). Cells were lysed by ultrasonication on ice and debris was removed by centrifugation (12,000×*g*, 20 min, and 4 °C). Lysed supernatants were heat-treated at 50 °C for 30 min and denatured proteins were removed by centrifugation (12,000×*g*, 20 min, and 4 °C). Proteins were finally purified by immobilized metal affinity chromatography. Briefly, the centrifugal supernatants were loaded individually onto a His-tag Ni-affinity resin (National Engineering Research Centre for Biotechnology, Beijing, China), which had been pre-equilibrated with binding buffer. After that, columns were washed with binding buffer to remove non-associative proteins. Fusion proteins that immobilized to the resin were thereby eluted with elution buffer (50 mM Tris–HCl, pH 7.5, 300 mM NaCl, and 150 mM imidazole). Purified recombinant proteins were confirmed by sodium dodecyl sulfate-polycrymide gel electrophoresis (SDS-PAGE) as described by Laemmli [40].

### Circular dichroism spectra

The CD spectra of Xyn10B-TM1, Xyn10B-TM2, Xyn10B-TM3 and Xyn10B-TM4 were performed on a circular dichroism spectrometer (JASCO J-810, Jasco Inc., Easton, MD). The protein was prepared with concentration of 0.2 mg/mL in NaH_2_PO_4_ buffer (20 mM, pH 7.5). The CD spectra were measured at room temperature over a range of 190–260 nm using a quartz cuvette of 1 mm path length, with a bandwidth of 1 nm and a scanning speed of 100 nm/min. The measurements were conducted in triplicate, and the data was corrected and analyzed by subtracting the buffer blank spectrum.

### Enzymatic activity screening and protein determination

The hydrolytic activity of recombinant constructs were screened, respectively, with various kinds of 1% (w/v) polysaccharides including BWX, SCX, OSX, CCX, Avicel, carboxymethyl cellulose, soluble starch, locust bean gum and chitosan in citrate buffer (50 mM sodium citrate, 300 mM NaCl, pH 6.0). Specific activity of xylanase was assayed by incubating appropriate amount of recombinant enzyme with 1% (w/v) xylan substrates in 100 μL reaction buffers for 2 min based on optimal pH value and temperature characters. Kinetic parameters of Xyn10B-TM1 were determined using different concentrations (0.1–1.0%, w/v) of xylan substrates in pH 6.0 citrate buffer at 65 °C. The amount of reducing sugars produced by recombinant constructs of Xyn10B were determined using *para*-hydroxybenzoic acid hydrazide (PHBAH) method with xylose as a standard [41]. One unit of xylanase activity was defined as the amount of enzyme needed to generate 1 μmol reducing sugar per minute under standard assay conditions. Protein concentrations were determined by Bradford method using bovine serum albumin (BSA) as standard. All the assays were performed in triplicate.

### Biochemical characterization of the recombinant Xyn10B truncated variants

For determination the temperature optimum of recombinant constructs, enzyme activity was measured as described above in 100 μL citrate buffer (50 mM sodium citrate, 300 mM NaCl, pH 6.0) at temperatures ranging from 40 to 95 °C for 2 min. The pH dependency of xylanase activity was analyzed with a variant of citrate buffer (50 mM sodium citrate, 300 mM NaCl, pH 4.0–6.5) and phosphate buffer (50 mM sodium phosphate, 300 mM NaCl, pH 6.5–8.5) at 65 °C for 2 min. Thermostability was monitored by incubating enzyme preparations at 60, 65, and 70 °C for time period of 0.5, 1, 2, 4, 6 and 12 h. Residual activity under optimal conditions was assayed respectively in triplicate with 0.5% (w/v) BWX as described above.

### Polysaccharides binding assay

The binding assay was conducted by adding 0.8 μg purified enzyme individually to 10 mg insoluble polysaccharides in 200 μL buffer. The reaction pH was controlled at 6.0 for Xyn10B-TM1 and Xyn10B-TM2 and 6.5 for Xyn10B-TM3 and Xyn10B-TM4. The enzyme-xylan mixtures were incubated in a vertical mixing apparatus at 4 °C for 1 h. The rest unbound enzymes in the supernatant after centrifugation (4000×*g*, 5 min, and 4 °C) were determined at 595 nm by Bradford method. The ratio (%) of binding protein to insoluble polysaccharides to the total protein presenting in the assay mixture was defined as the relative binding. Polysaccharides tested were insoluble OSX, insoluble BWX and Avicel. Insoluble xylan was prepared by washing with water for three times, and the pellet was dried and used as insoluble fractions. All the binding assays were performed in triplicate with BSA as a control.

### Substrate hydrolysis patterns of the recombinant Xyn10B truncated variants

To evaluate the hydrolysis patterns or substrate specificity of the different Xyn10B constructs, hydrolysis products of various kinds of xylan were quantified through reducing sugar assay and high performance liquid chromatography (HPLC) analysis. After pre-incubation, 200 μL citrate buffer, containing 10 mg xylan and 25 μg enzyme were incubated at 65 °C for 24 h. The reaction pH was controlled at 6.0 for Xyn10B-TM1 and Xyn10B-TM2, and 6.5 for Xyn10B-TM3 and Xyn10B-TM4. Each control reaction was performed under the same experimental conditions except adding heat denatured enzyme. After reaction, hydrolysis products were instantaneously centrifuged and analyzed for the whole reducing sugar. The amount of xylose produced by Xyn10B truncated derivatives was determined by HPLC equipped with a Hi-PlexH exclusion column (300 × 7.7 mm, Agilent Technologies, United Kingdom) and a refractive index detector (LC-20AT, Shimadzu Corp., Japan). Column temperature was maintained at 65 °C with injection volume of 10 μL. Milli-Q filtered 0.005 M H_2_SO_4_ was used as mobile phase at a flow rate of 0.6 mL/min. Xylose was applied as a standard. The highest activities for each xylan substrate were respectively defined as 100%. All of the assays were performed for three times.

## Additional files

**Additional file 1: Fig. S1.** Multiple amino acid sequence alignment of the GH10 catalytic domain of *C. lactoaceticus* Xyn10B and other GH10 xylanases. **Fig. S2.** SDS-PAGE analysis of *C. lactoaceticus* Xyn10B truncated variants.

**Additional file 2: Table S1.** CD spectra for *C. lactoaceticus* Xyn10B truncated variants.

## Data Availability

All data generated or analyzed during the study are included in this published article and its additional file.

## Abbreviations

GH: glycoside hydrolase
CBM: carbohydrate-binding module
CD: catalytic domain
SLH: S-layer homology
p*I*: isoelectric point
CD: circular dichroism
BWX: beechwood xylan
OSX: oat spelt xylan
SCX: sugar cane xylan
CCX: corncob xylan
LB: Lysogeny Broth
IPTG: isopropyl-β-d-thiogalactopyranoside
SDS-PAGE: sodium dodecyl sulfate-polycrymide gel electrophoresis
PHBAH: para-hydroxybenzoic acid hydrazide
BSA: bovine serum albumin
HPLC: high performance liquid chromatography
