# Supplementary material for: The extracellular endo-β-1,4-xylanase with multidomain from the extreme thermophile Caldicellulosiruptor lactoaceticus is specific for insoluble xylan degradation
Source: Biotechnol Biofuels. 2019 Jun 8;12:143. doi: 10.1186/s13068-019-1480-1 (PMC6556019; doi:10.1186/s13068-019-1480-1)
Supplement: Supplementary file 1 — Additional file 1: Fig. S1. Multiple amino acid sequence alignment of the GH10 catalytic domain of C. lactoaceticus Xyn10B and other GH10 xylanases. Fig. S2. SDS-PAGE analysis of C. lactoaceticus Xyn10B truncated variants. [file 13068_2019_1480_MOESM1_ESM.docx]

**The extracellular endo-β-1,4-xylanase with multidomain from the extreme thermophile *Caldicellulosiruptor lactoaceticus* is specific for insoluble xylan degradation**

Xiaojing Jia^1,2,3^ and Yejun Han^2*^

^1^ *Beijing Advanced Innovation Center for Food Nutrition and Human Health, Beijing Technology & Business University, Beijing 100048, China*

^2^ *National Key Laboratory of Biochemical Engineering, Institute of Process Engineering, Chinese Academy of Sciences, Beijing 100190, China*

^3^ *University of Chinese Academy of Sciences, Beijing 100049, China*

*Address correspondence to: Yejun Han

Correspondence: [yjhan@ipe.ac.cn](mailto:yjhan@ipe.ac.cn) or [yejunhan09@gmail.com](mailto:yejunhan09@gmail.com)

#

**Fig. S1** Multiple amino acid sequence alignment of the GH10 catalytic domain of *C. lactoaceticus* Xyn10B and other GH10 xylanases.

Sequence alignment was computed on <http://www.genome.jp/tools/clustalw/> and depicted by ESPrit 3.0: <http://espript.ibcp.fr/ESPript/cgi-bin/ESPript.cgi>. Identical and similar amino acid residues are enclosed in black boxes and open boxes, respectively. The residues Glu619, Glu734, and Trp790 are marked by black arrows. The alignment includes the GH10 catalytic domain of *Thermoanaerobacterium saccharolyticum* Jw/sl-ys485 Tsxyla [PDB: 3W24_A], *Clostridium stercorarium* F9 Xylanase B [PDB: 2DEP_A], *Geobacillus stearothermophilus* Ixt6 [PDB: 1N82_A], and *Bacillus stearothermophilus* Xt6 [PDB: 1HIZ_A], and *Paenibacillus barcinonensis* Xynb [PDB: 3EMC_A].

# Fig. S2 SDS-PAGE analysis of *C. lactoaceticus* Xyn10B truncated variants
