# Supplementary material for: The extracellular endo-β-1,4-xylanase with multidomain from the extreme thermophile Caldicellulosiruptor lactoaceticus is specific for insoluble xylan degradation
Source: Biotechnol Biofuels. 2019 Jun 8;12:143. doi: 10.1186/s13068-019-1480-1 (PMC6556019; doi:10.1186/s13068-019-1480-1)
Supplement: Supplementary file 2 — Additional file 2: Table S1. CD spectra for C. lactoaceticus Xyn10B truncated variants. [file 13068_2019_1480_MOESM2_ESM.docx]

# Table S1. CD spectra for *C. lactoaceticus* Xyn10B truncated variants ^a^

|  | Xyn10B-TM1 | Xyn10B-TM2 | Xyn10B-TM3 | Xyn10B-TM4 |
| --- | --- | --- | --- | --- |
| α-helix (%) | 25.1 ± 0.5 | 19.9 ± 0.3 | 47.6 ± 0.8 | 34.7 ± 0.5 |
| β-sheet (%) | 32.5 ± 0.1 | 25.7 ± 0.2 | 0.0 ± 0.1 | 25.6 ± 0.3 |
| β-turn (%) | 15.4 ± 0.2 | 21.2 ± 0.1 | 9.6 ± 0.1 | 14.4 ± 0.2 |
| Random coil (%) | 27.0 ± 0.1 | 33.2 ± 0.1 | 42.7 ± 0.2 | 25.3 ± 0.2 |

^a^ Data are presented as means ± standard errors.
